# Anthony (Tony) McMichael: 1942–2014

**DOI:** 10.1289/ehp.122-A290

**Published:** 2014-11-01

**Authors:** Kristie L. Ebi, Colin Butler

Anthony (Tony) McMichael died 26 September 2014 in Canberra, Australia, at the age of 71. McMichael graduated in medicine from the University of Adelaide in 1967 and earned a PhD from Monash University in 1972, where he was the first doctoral student in epidemiology. He recognized and coined the term “healthy-worker effect” while doing postdoctoral research at the University of North Carolina at Chapel Hill. After returning to Australia, he worked in the field of nutrition and health with the Commonwealth Scientific and Industrial Research Organisation (CSIRO) before being recruited as the Foundation Chair in Occupational and Environmental Health at the University of Adelaide. He was a professor of epidemiology at the London School of Hygiene and Tropical Medicine from 1994 to 2001 and directed the Australia National University National Centre for Epidemiology and Population Health from 2001 to 2006. McMichael held a National Health and Medical Research Council Australia Fellowship, Australia’s most prestigious award for excellence in the fields of health and medical research. In 2011, he was made an Officer of the Order of Australia and was elected to the U.S. National Academy of Sciences. At his death, he held honorary positions at the University of Copenhagen and the London School of Hygiene and Tropical Medicine, and he was a long-standing advisor to the World Health Organization. He also was a Fellow at Chatham House on Global Health Security and a Fellow of the Australian Academy of Technological Sciences.

McMichael was a pioneer in developing research on the health risks and burdens of global climate change and other large-scale environmental changes. His book *Planetary Overload: Global Environmental Change and the Health of the Human Species* (1993) established global environmental change as a public health issue. He worked tirelessly to promote understanding of the challenges humans are creating through patterns of development, paying particular attention to the risks for highly vulnerable populations. He urged epidemiologists to, in his words, escape “the prison of the proximate”; that is, in the study of population health, epidemiologists should look beyond individual-level risk factors to the roles of social and ecologic systems. In other work, he examined how the long-term record of climatic trends and fluctuations affected human health, well-being, and survival. He was also involved in developing approaches to study the roles of urban settlements, food production and consumption patterns, transportation, and energy use, among others, as determinants of environmental sustainability.

McMichael made significant contributions across a wide range of public health issues, including smoking, cancer, nutrition, infectious diseases, and the effects of lead on cognitive development. In an eminent and productive career, he published more than 300 peer-reviewed papers and 160 book chapters, coedited 9 books, and wrote 2 books, with a third nearly complete. McMichael retired from Australia National University in 2012. That same year, a *Festschrift* was held to commemorate his career; the associated book (*Health of People, Places and Planet. Reflections Based on Tony McMichael’s Four Decades of Contribution to Epidemiological Understanding*) will be published in 2015.

Upon meeting McMichael, one would not perceive that he was a scientific giant, providing insights that will continue to inform research and policy for years to come. He was the most humble and gracious of men, and a kind and thoughtful friend and mentor to many people worldwide. He willingly answered questions, shared references and slides, and provided guidance on epidemiological approaches for studying complex issues.

He had a delightful sense of humor. The opening of his keynote address to the International Epidemiological Association’s 20th World Congress on Epidemiology in Anchorage, Alaska, in August 2014 was typical: After graciously thanking his hosts for the invitation, he looked at his notes and said he should have listened to his wife and cleaned his glasses.

Tony McMichael’s scientific contributions, although legendary, do not give a sense of who he was—and that is what everyone who had the privilege of working with him will remember.

McMichael is survived by his wife Judith Healy, his daughters Anna and Celia, four grandchildren, and his brothers Philip and Robert.

